# Efficacy of an Interactive Life Simulation Gaming App on Improving HIV Testing Uptake Among Adolescents and Young Adults at Risk for HIV: Results of a Randomized Controlled Trial

**DOI:** 10.2196/82297

**Published:** 2026-09-04

**Authors:** Amanda D Castel, Irene Kuo, Adam Ciarleglio, Lucas Kauzlarich, Constance Trexler, Sachi Banerji, Tamara C Moscovich, John C Banas, Constanza Carney, Callen Shaw, Daniel Greenberg

**Affiliations:** 1 Department of Epidemiology, Milken Institute School of Public Health, The George Washington University, 950 New Hampshire Ave NW, 5th floor, Washington, DC, 20052, United States, 1 2029948325, 1 2029940082; 2 Department of Biostatistics and Bioinformatics, Milken Institute School of Public Health, The George Washington University, Washington, DC, United States; 3 Division of Adolescent Medicine, Children's National Medical Center, Washington, DC, United States; 4 School of Medicine and Health Sciences, The George Washington University, Washington, DC, United States; 5 Media Rez, LLC, Washington, DC, United States

**Keywords:** intervention, randomized trial, HIV testing, interactive gaming, pre-exposure prophylaxis, adolescents, young adults, prevention, efficacy

## Abstract

**Background:**

Adolescents and young adults account for a disproportionate number of new HIV diagnoses in the United States due to missed opportunities for education, testing, and prevention.

**Objective:**

We tested the efficacy of an interactive life-simulation gaming intervention to provide HIV education and improve HIV testing uptake and preventive services among youths ages 13‐24 years in the Washington, DC area.

**Methods:**

We conducted a parallel randomized controlled trial testing the efficacy of an HIV-focused gaming intervention app to a control app in increasing HIV testing uptake. Participants were recruited via social media and peer referral. Eligible participants were randomized 1:1 by age (13‐17 vs 18‐24 years) and sexual orientation (lesbian, gay, bisexual, or “other” vs heterosexual). Participants accessed their assigned app for 3 months and completed baseline, 1-, 3-, and 6-month surveys. The primary outcome was self-reported HIV testing within 6 months after enrollment; secondary outcomes included intent to test for HIV, HIV knowledge, and intention to start pre-exposure prophylaxis (PrEP). Analyses were conducted as intent-to-treat and stratified by sexual orientation.

**Results:**

From November 2023 to May 2024, we randomized 309 participants (intervention: n=156; control: n=153) with 80% and 88% retention at 6 months, respectively. Among the analyzed participants (intervention: n=150; control: n=149), the mean age was 20.7 (SD 1.84) years, 49% (145/299) were women, and 34% (103/299) were non-Hispanic White. Overall, 53% (158/299) self-reported as gay, lesbian, bisexual, or “other” sexual orientation. A total of 48% (144/299) had previously tested for HIV. Compared to the control app, game participants were 32% less likely to test for HIV within 6 months (relative risk 0.68, 95% CI 0.47‐0.98). The two groups showed no statistically significant differences regarding their intention to get tested for HIV or to start PrEP. A significantly higher difference in mean HIV knowledge scores at 3 months (0.99, 95% CI 0.33‐1.65; *P*<.01) and 6 months (0.81, 95% CI 0.14‐1.48; *P*=.02) was observed in the game arm compared to the control arm. Additionally, heterosexual participants in the game arm had statistically significant increases in PrEP intention (0.53, 95% CI 0.05‐1.00; *P*=.03) across all time points compared to controls.

**Conclusions:**

Our interactive gaming app was less effective than an information control app in increasing HIV testing and changing testing and prevention intentions among adolescents and young adults. In the game arm, we did observe increased HIV knowledge and increased PrEP intention among heterosexual adolescents and young adults. Few life-simulation apps integrating HIV testing and prevention features have been rigorously tested among adolescents and young adults. Our efficacy findings suggest that while these interventions are feasible and acceptable to adolescents and young adults, they require additional tailoring to improve their reach, use, and potential effectiveness in real-world settings.

## Introduction

### Background and Rationale

Adolescents and young adults ages 13‐24 years account for 18% of new HIV infections in the United States, with racial and ethnic minority groups and men who have sex with men being disproportionately impacted [1]. Additionally, an estimated 50% or more of youths in the United States are unaware they are living with HIV, indicating that there are critical gaps in getting adolescents and young adults tested for HIV [2,3]. Accordingly, national initiatives such as the US National HIV/AIDS Strategy and the Ending the HIV Epidemic initiative have focused on reducing HIV incidence in this key population through efforts such as improved self-assessment of HIV risk, increased uptake of HIV prevention efforts, and use of routine HIV testing [4-6].

In Washington, DC, a priority jurisdiction for Ending the HIV Epidemic efforts, in 2023, 36% of new HIV diagnoses were among persons ages 13‐29 years, making this the leading age group for new diagnoses [7]. Adolescents and young adults in DC have high rates of gonorrhea and chlamydia with 29% and 45% of diagnoses in 2023, respectively, occurring among persons ages 13‐24 years [7]. These high rates of sexually transmitted infections (STIs) are likely a result of engaging in condomless sex; thus, adolescents and young adults may also be exposing themselves to HIV [8]. Data from the 2023 Youth Risk Behavioral Survey conducted in DC among high school students further support these risky behaviors, with 82%‐84% of sexually active youths reporting never being tested or not sure if they were ever tested for an STI and 76%‐80% never being tested or not sure if they were ever tested for HIV [9]. Finally, these data highlight the need for increased education around HIV prevention, with as many as 39% of heterosexual and 48.9% of lesbian, gay, and bisexual high school students not receiving HIV education at school [9].

Despite engaging in these risky behaviors, and initiatives focused on routine testing among adolescents and young adults, adolescents and young adults do not partake in routine HIV testing, which results in missed opportunities for HIV education, prevention, and possibly early diagnosis [10-16]. Barriers to testing include lack of provider screening, lack of self-perceived risk, lack of knowledge regarding HIV and HIV prevention services such as pre-exposure prophylaxis (PrEP), and difficulty identifying and accessing HIV testing sites [16-21]. These data suggest that there are significant gaps in our ability to educate youths on how to prevent HIV, engage in safe sexual behaviors, and access resources such as HIV testing and PrEP [12,17,22].

The use of technology-based interventions to address HIV among adolescents and young adults has emerged over the last decade [23-25]. Moreover, with the pervasiveness of mobile phones and use of social media among adolescents and young adults, digital gaming is a promising approach to facilitate the delivery of health-related information, particularly as it relates to HIV. A systematic review conducted in 2015 identified 55 smartphone, internet, and web-based interventions addressing the HIV care continuum, of which only 5 used gaming approaches [24]. More recent reviews have identified more than 30 game-based interventions focused on HIV prevention [26]. For example, an increasing number of digital game–based interventions, or serious games, have been developed to address various steps along the HIV care continuum, including to promote sexual health literacy [27,28], HIV risk assessment and reduction [29-31], HIV prevention and testing [32-34], as well as PrEP adherence [35]. Although some of these programs have demonstrated preliminary acceptability and feasibility, results from several of these trials are forthcoming, and there is limited research on their efficacy in increasing HIV testing among adolescents and young adults [33,36-39].

Evidence also suggests that when people practice new behaviors in virtual environments, they acquire new skills that they are more likely to apply in real-life situations [30,31,40-43]. Additionally, these games can be used to assess varying levels of exposure to an intervention and have the potential for sustained exposure [30,31].

### Objectives

Given the high rates of HIV among adolescents and young adults, the need to increase HIV testing and access to prevention interventions, and the pervasive use of technology, more specifically gaming, we sought to test the efficacy of a theoretically based interactive and entertaining dating-and-life simulation game with the goal of increasing HIV knowledge and risk assessment and facilitating access to HIV testing and PrEP. Our intervention was grounded in social cognitive theory [44] and the health belief model [45] and iteratively informed and designed through focus groups and pilot testing by adolescents and young adults [46]. The final intervention product was designed to offer adolescents and young adults personally tailored risk messages and support their accessibility to HIV testing and prevention services through an innovative gaming approach [47]. In this analysis, our objective was to assess the efficacy of the interactive, life-simulation game to increase HIV testing, knowledge, and prevention, and reduce risk behaviors among adolescents and young adults ages 13‐24 years compared to a control app that provided static information on HIV prevention, care, and treatment.

## Methods

### Participant Recruitment

Between November 2023 and May 2024, participants were recruited using a combination of community-based outreach and social media advertisements. Targeted advertisements were developed and placed on social media sites (ie, Facebook, Instagram, Twitter, Snapchat, and YouTube). In addition, advertisements were placed at local community-based organizations, local colleges and universities, and 2 local public health clinics (DC Health and Wellness Center and the Montgomery County Maryland Health Center). Potential participants were able to scan a QR code or click on an advertisement to complete an online screener that assessed their age, gender, sexual behaviors and orientation, state of residence, and availability of a reliable cell phone. Peer referral was also used, where eligible participants were provided with unique links to share with up to 3 of their peers. Participants received an additional US $10 incentive for each peer recruited who participated in the trial. REDCap was used to generate peer referral links, screen potential participants, and record and manage the eligibility screener surveys for peer-recruited participants [48,49].

Eligible participants were ages 13 to 24 years, self-reported being HIV negative or unknown status, self-reported ever being sexually active (inclusive of oral, anal, or vaginal sex), were residents of the DC metropolitan region (DC, Southern Maryland, and Northern Virginia) for at least the next 6 months, were able to provide informed consent, were able to complete all study procedures in English, and had their own mobile phone to be used for the study duration. To reduce the risk of fraud due to online screening, all participants meeting eligibility criteria were contacted by telephone by a member of the study team to confirm their responses and eligibility for the study.

A waiver of parental consent was obtained for participants aged 13 to 17 years as well as a waiver of written informed consent. After participants were screened for eligibility and fraud, they were provided with a link to REDCap to provide electronic consent or assent.

There was no public involvement in the design or reporting of the trial. A separate group of adolescents and young adults helped inform the intervention but were not involved in the conduct of the trial [47].

### Trial Design

This study has been reported in accordance with the CONSORT (Consolidated Standards of Reporting Trials) 2025 checklist (Checklist 1) and the CONSORT-eHEALTH (Consolidated Standards of Reporting Trials of Electronic and Mobile Health Applications and Online Telehealth) checklist for randomized controlled trials [50,51].

### Study Arm Randomization

Once verified and consented, participants were randomized 1:1 to 1 of 2 study arms for a 3-month period: (1) the intervention arm, in which participants had unlimited access to the interactive game, as well as a Centers for Disease Control and Prevention (CDC)–supported HIV risk estimator tool [52], and HIV and PrEP locators [53], both of which were embedded within the game; or (2) the control arm, in which participants had access to a static HIV educational app curated from existing resources including the U.S. CDC website on HIV, the CDC HIV risk estimator tool, and the HIV and PrEP locator websites. Participants were randomized in blocks of 8, stratifying by age (13-17 and 18-24 years) and sexual orientation (lesbian, gay, bisexual, or “other” vs heterosexual), using a random number generator for assignment. Randomization blocks were set up using R (R Foundation for Statistical Computing) by the study biostatistician (AC) and applied through REDCap by the study research coordinators (CC and LK). The research coordinators did not have access to the randomization allocation sequence. Once enrolled, participants completed a baseline survey through REDCap and received a link to download either the game or control app to their personal smartphone. There was no blinding after assignment or allocation concealment.

### Intervention Condition and Delivery

**Figure 1.**
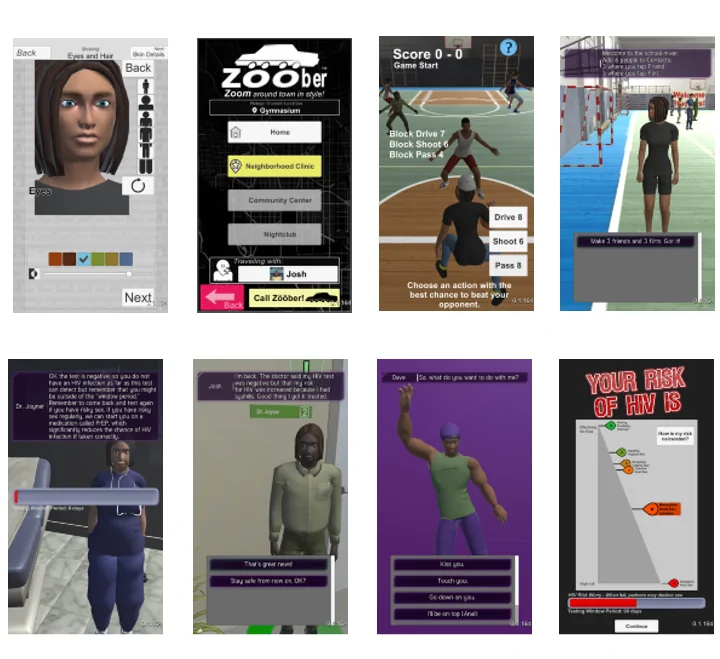
Sample images of life-simulation gaming app intervention.

The game intervention included a dating and life simulation game that offered a personalized avatar and multiple mini scenes in which participants could complete mini-games or quests (eg, basketball gymnasium, community center, neighborhood clinic waiting room, and nightclub), meet and interact with friends, engage in interactive virtual dating, and identify potential sexual partners. After downloading the app to their personal mobile phones, participants had the opportunity to create their personal avatar. They were able to progress through the game at their own pace, playing the game for as little or as long as they desired. They had 18 opportunities to learn about HIV prevention (eg, condom use, postexposure prophylaxis, PrEP, and STI testing), HIV testing and treatment (eg, routes of transmission and how and where to get testing), stigma, risk reduction, and how PrEP works (“HIV learning content”) through interactions with in-game characters (Figure 1). Participants could engage in in-game dialogue with characters in the game to facilitate their ability to learn about HIV. Through prespecified dialogue, the participant could choose to engage in a hypothetical virtual sexual encounter. Anytime a participant had a hypothetical virtual sexual encounter (not graphically depicted) with an in-game sexual partner, they could make choices regarding their sexual behaviors and risks (eg, oral, anal, or vaginal sex, use of condoms, or PrEP). Once their choices were made, their risk for HIV was calculated using an integrated version of the CDC HIV risk estimator, and a link to an HIV testing and PrEP locator was available. Participants could choose to click on a link to be connected to a real-life HIV testing or PrEP site of their choice. Once downloaded, participants had access to the game for a 3-month period, with up to 30 hours of content available. Participants were asked to opt in to receive up to 4 notifications to encourage them to engage with the game. Notifications occurred after the first 7 days of no game activity and at 30, 60, and 90 days after enrollment.

### Control Condition Development and Delivery

**Figure 2.**
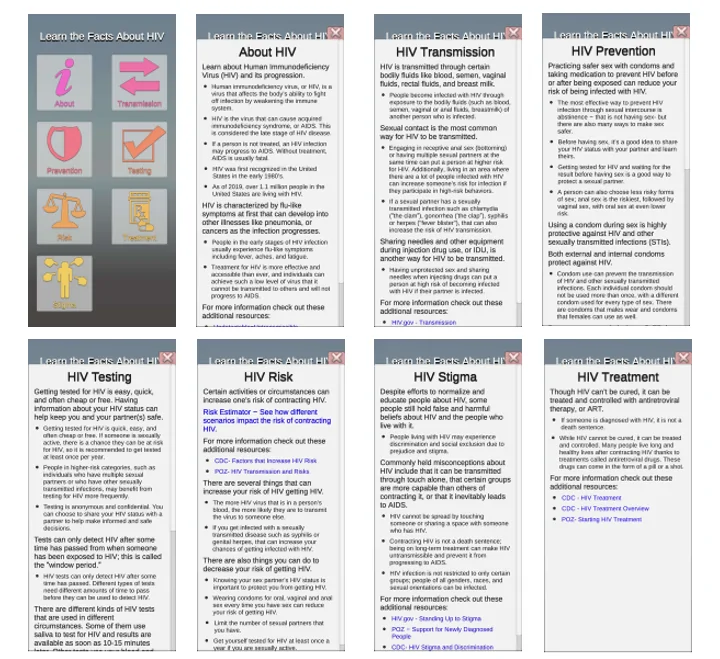
Sample images of HIV informational app content.

Prior to the rollout of the intervention, the game development team (Media Rez), along with the clinical research team, developed a control app that included the same basic HIV content included in the game intervention, as mentioned earlier (eg, data on HIV transmission, prevention information, and information on HIV testing and PrEP). It also included links for additional information as well as a link to the CDC HIV risk estimator and HIV testing and PrEP locators (Figure 2). After providing informed consent and completing a baseline survey through REDCap, participants randomized to the control arm received a link to download the informational app (both iOS and Android compatible) and had unlimited access to the app for a 3-month period. Participants were asked to opt in to receive up to 4 notifications to encourage them to engage with the control arm app. Notifications occurred after the first 7 days of no control app openings and at 30, 60, and 90 days after enrollment.

### Data Collection and Management

The primary outcome for the study was being tested for HIV within the 6-month period after enrolling in the study. HIV testing was measured by participant self-report at the 6-month behavioral survey. Secondary outcomes included participant self-reported HIV and PrEP knowledge, intention to test for HIV, intention to start PrEP, condom self-efficacy, and self-perceived risk. These outcomes were measured using previously validated scales at each time interval.

Data were collected through REDCap surveys at baseline, 1, 3, and 6 months after enrollment. Surveys included questions regarding self-reported sexual and HIV risk behaviors, self-perceived risk for HIV [54], barriers to HIV testing, reported HIV testing and PrEP uptake, behavioral intentions, which included intent to test for HIV [55], intent to start PrEP [56], and reduce risky behaviors, self-efficacy [57], game use, interest, acceptability, and usability [58].

To minimize nonresponse rates and social desirability bias with respect to self-reported data, all surveys were sent to participants via a web link using the REDCap system. Participants received US $30 for completion of the baseline survey, US $25 for the 1-month, US $25 for the 3-month, and US $40 for the 6-month survey.

### Game and Control App Paradata and Game Usability, Acceptability, and Satisfaction

We monitored the intervention and control arm apps in real time using game data and paradata, that is, supplementary data to quantify technology use [59,60]. Paradata collected for participants in the intervention arm included length of time using the game, HIV-related game content accessed, and use of locators. For those in the control arm, use of the app, including the number of times the app was opened, and clicks on external content were collected for each participant. We also assessed game and control app usability, acceptability, and satisfaction. The System Usability Score (SUS) was used to measure game usability, with possible scores ranging from 0 to 100, with a mean score of 68 or higher indicating above-average usability [53]. Selected items from the MAKE (Motivation, Attitude, Knowledge, and Engagement) measure were used to assess game acceptability, which included domains to measure participants’ motivation, attitude, knowledge, and engagement with the game or control arm [61]. Each domain was scored with a total possible score of 160. Game satisfaction was measured using an 8-item scale with responses ranging on a Likert scale of 1 to 4, with a total possible range of scores from 8 to 32 [62].

### Retention Protocol

The study research assistants and coordinators (JB, SB, CC, LK, and TM) were in contact with participants throughout the follow-up period to support downloading of the app or game, provide technical support, and provide study reminders and updates. Participants were sent a reminder of an upcoming survey 2 weeks and 1 week prior to their survey date. If participants did not complete the follow-up survey, the link was resent, and the participant was contacted via email or text message 24, 48, and 72 hours after being sent the initial email or text notification to remind them to complete it. If there was no response, the study research assistants and coordinators (JB, SB, CC, LK, and TM) contacted the participant to confirm receipt of the link and to assist them in completing it. Participants had up to 2 weeks to complete each survey. If a participant was nonresponsive after 10 attempts, the participant was considered lost to follow-up.

### Statistical Methods

We described the study sample’s clinical, demographic, and sexual behavior characteristics at baseline, both overall and stratified by treatment arm. We reported mean (SD) or median (IQR) for numerical variables and frequency (proportion) for categorical variables.

For the primary outcome of HIV testing within 6 months, we reported the frequency (proportion) of participants who self-reported being tested for HIV within 6 months in each treatment arm, along with the corresponding risk ratio, 95% CI, and *P* value from the corresponding Pearson chi-square test. These same statistics were also reported for the secondary outcomes of intent to test (coded as “likely” if score was 4 or higher and “unlikely” if score was 3 or lower) and PrEP initiation at 6 months [55]. Since some participants were missing either one or more of these outcomes, we also conducted a sensitivity analysis using multiple imputation by chained equations [63] to impute the missing data and then assessed treatment effect using the imputed data. A full description of the procedure and results is provided in Multimedia Appendix 1. Similar analyses, stratified by sexual orientation, are provided in Multimedia Appendix 1.

For the HIV knowledge and intent scores, we fit linear mixed effects models with each score as the outcome and with treatment arm, time (coded as a 4-level categorical variable with baseline as the reference level and dummy variables for 1, 3, and 6 months), and the interaction between treatment arm and time as predictors. A participant-specific random intercept was also included to account for correlation between repeated observations from the same individual. We conducted a likelihood ratio test (LRT) using Kenward-Roger denominator degrees of freedom to test for a significant interaction between treatment arm and time. If the LRT *P* value was less than .05, we computed the model-based contrasts between the 2 treatment arms at each time point along with the corresponding 95% CIs and Wald test *P* values. If the LRT *P* value was greater than or equal to .05, we dropped the interaction terms and refitted the model with only the main effects of treatment arm and time and reported the model-based estimate for treatment effect and corresponding 95% CI and Wald test *P* value. Similar analyses, stratified by sexual orientation, are provided in Multimedia Appendix 1. In the exploratory analyses, we also conducted each analysis for the primary and secondary outcomes, stratified by sexual orientation (heterosexual vs non-heterosexual).

We computed mean and SD for the SUS, game and app satisfaction scores, and the MAKE scale measures of game-based learning. We also computed descriptive summaries for selected paradata measures. These results are provided in Multimedia Appendix 1.

A significance level of .05 was used throughout. All analyses were conducted using R (version 4.2.2) and the following packages were used: *lme4*, *lmerTest*, *emmeans*, and *mice* [63-66].

There were no changes to the trial protocol after enrollment began with respect to eligibility criteria, the interventions, outcomes, sample size, or analysis methods. The original randomization process was changed from a block size of 6 to a block size of 8; however, the treatment allocation ratio remained 1:1, allocation concealment was maintained, and the randomization sequence was generated prior to enrollment. Despite this change, the final treatment groups remained well balanced with respect to sample size and baseline characteristics, particularly on the stratification characteristics of age and sexual orientation.

### Ethical Considerations

All study activities were reviewed and approved by the George Washington University Institutional Review Board (IRB # NCR191708) and the Children’s National Hospital IRB. IRB approval included a waiver of parental permission and a waiver of written consent for participants younger than 18 years of age per 45 CFR 46.404. Privacy and confidentiality protections were put in place accordingly to safeguard the identity of participants, and all data were deidentified for analysis. No identification of individual participants was used in any images or supplementary materials. The informed consent forms were revised in March 2024 to include that there may be risks of discomfort from playing the game, as it discussed sensitive topics regarding sexual behaviors. Participants could receive up to US $30 for peer referrals and up to US $120 for participation in all study activities. The trial was registered prospectively, and the first participant was enrolled on November 14, 2023.

### Harms

Harms and unintended effects to study participants were monitored by study staff. There were 2 participants who withdrew from the study. One participant reported being uncomfortable with the sexual content in the game, prompting a change in the informed consent language. No other harms were reported by participants during the study period.

### Sample Size

We conducted a power analysis to determine the appropriate sample size to be able to determine the efficacy of the intervention. We determined that a sample with approximately 300 participants (150 per arm) would achieve >80% power to detect a moderate effect corresponding to a difference of at least 13 percentage points (equivalent to a relative risk [RR] of 2.3 assuming a 10% prevalence of past 4‐6 months HIV testing in the control group) in HIV testing between the intervention and control groups after 6 months using a 2-sided test at a 5% significance level and assuming a 16% attrition rate after 6 months.

## Results

### Participant Recruitment, Enrollment, and Retention

**Figure 3.**
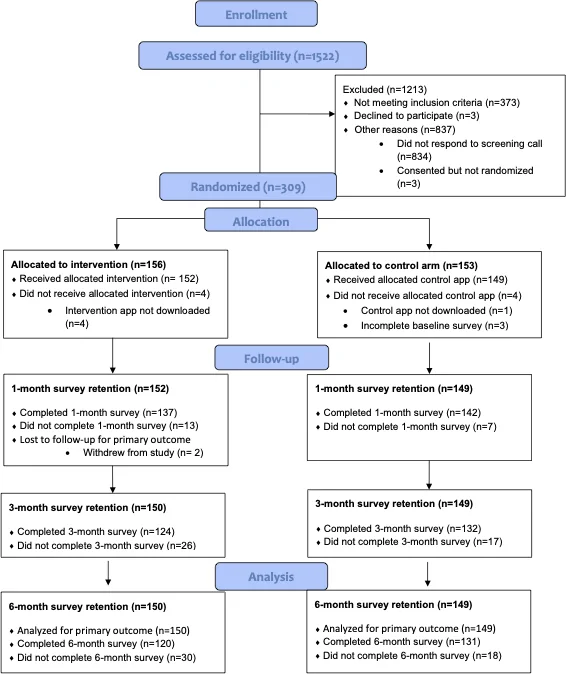
Study eligibility, enrollment, and retention.

Over a 6-month period, we screened 1522 people for study eligibility, of whom 1213 (80%) were ineligible for reasons including not meeting inclusion criteria (n=373), not responding to the fraud check screening call (n=834), refusing to participate (n=3), or being consented but not randomized (n=3; Figure 3). Of the remaining 309 (20%) who were eligible and randomized, 156 (51%) participants were assigned to the intervention arm and 153 (50%) assigned to the control arm. A total of 299 participants successfully downloaded either the game or intervention app and completed baseline surveys. Participants were primarily recruited through social media (Instagram, Facebook, or Snapchat, 84%) followed by peer-referral (16%). Study retention at 1, 3, and 6 months in the intervention arm was 90% (137/152), 83% (124/150), and 80% (120/150), respectively. Study retention at 1, 3, and 6 months in the control arm was 95% (142/149), 89% (132/149), and 88% (131/149), respectively. Our final 6-month analytic end-point analysis included 150 intervention arm participants and 149 control arm participants who completed at least 1 study follow-up survey.

### Baseline Participant Demographics

Participants were a mean age of 20.7 (SD 1.84) years, with 8 (3%) participants being younger than 18 years of age (Table 1). Approximately 19% (56/299) were Hispanic, 20% (59/299) were non-Hispanic Asian, 19% (58/299) were non-Hispanic Black, and 34% (103/299) were non-Hispanic White. Women accounted for 49% (145/299), 41% (121/299) identified as men, 8% (23/299) identified as nonbinary, and 3% (4/299) identified as transgender. Participants were 46% (137/299) DC residents, 45% (135/299) had completed some college or technical school, and 58% (173/299) were employed. With respect to sexual orientation, 47% (141/299) reported being heterosexual and 47% (141/299) as gay, lesbian, or bisexual, with 6% (7/299) reporting “other” sexual orientation. Almost half (144/299, 48%) had previously tested for HIV, with 17% (52/299) reporting being tested within the prior 3 months.

**Table 1.** Participant demographics stratified by study arm.

	Total (n=299)	Control app (n=149)	Game intervention (n=150)
Age (years), mean (SD)	20.73 (1.84)	20.81 (1.84)	20.65 (1.84)
Race or ethnicity, n (%)			
Hispanic any race	56 (19)	24 (16)	32 (21)
Non-Hispanic Asian only	59 (20)	27 (18)	32 (21)
Non-Hispanic Black only	58 (19)	34 (23)	24 (16)
Non-Hispanic—multiracial	19 (6)	9 (6)	10 (7)
Non-Hispanic White only	103 (34)	52 (345)	51 (34)
Other	4 (1)	3 (2)	1 (1)
Gender, n (%)			
Men	121 (401)	67 (45)	54 (36)
Women	145 (49)	71 (48)	74 (49)
Transgender woman	4 (1)	0 (0)	4 (3)
Transgender man	4 (1)	1 (1)	3 (2)
Nonbinary	23 (8)	9 (6)	14 (9)
Other	2 (1)	1 (1)	1 (1)
Residence, n (%)			
DC	137 (46)	75 (50)	62 (41)
MD	85 (28)	47 (32)	38 (25)
VA	77 (26)	27 (18)	50 (33)
Education, n (%)			
1‐8 grade	2 (1)	2 (1)	0 (0)
9‐11 grade	9 (3)	3 (2)	6 (4)
12 or GED^a^	58 (19)	27 (18)	31 (21)
Some college or technical school	135 (45)	72 (48)	63 (42)
Bachelor degree	79 (26)	34 (23)	45 (30)
Any postgraduate	15 (5)	10 (7)	5 (3)
Prefer not to answer	1 (0)	1 (1)	0 (0)
Employed (yes), n (%)	173 (58)	86 (58)	87 (58)
Recruitment source, n (%)			
Snapchat	3 (1)	1 (1)	2 (1)
Facebook	4 (1)	2 (1)	2 (1)
Instagram	243 (81)	121 (81)	122 (81)
Children’s National Hospital	1 (0)	1 (1)	0 (0)
Friend	48 (16)	24 (16)	24 (16)
Mobile phone type, n (%)			
iPhone	262 (88)	130 (87)	132 (88)
Android	36 (12)	19 (13)	17 (11)
Other	1 (0)	0 (0)	1 (1)
Tobacco products (yes), n (%)	119 (40)	58 (39)	61 (41)
Alcoholic beverages (yes), n (%)	256 (86)	127 (85)	129 (86)
Cannabis (yes), n (%)	193 (65)	105 (71)	88 (59)
Other substance use (yes), n (%)	73 (24)	37 (25)	36 (24)
Sexual orientation			
Heterosexual	141 (47)	70 (47)	71 (47)
Lesbian, gay, bisexual	141 (47)	70 (47)	71 (47)
Other	17 (6)	9 (6)	8 (5)
Prior HIV test (%), n (%)			
No	132 (44)	58 (39)	74 (49)
Yes	144 (48)	76 (51)	68 (45)
Do not know	23 (8)	15 (10)	8 (5)
Most recent HIV test, n (%)			
0‐3 months	52 (17)	29 (20)	23 (15)
4‐6 months	37 (12)	17 (11)	20 (13)
7‐9 months	14 (5)	8 (5)	6 (4)
10‐12 months	14 (5)	5 (3)	9 (6)
>12 months	27 (9)	17 (11)	10 (7)
N/A^b^	155 (52)	73 (49)	82 (55)
Likely to test for HIV in the next 6 months, n (%)			
Extremely unlikely	71 (234)	34 (23)	37 (25)
Very unlikely	55 (18)	26 (17)	29 (19)
Somewhat unlikely	72 (24)	36 (24)	36 (24)
Somewhat likely	59 (20	31 (21)	28 (19)
Very likely	20 (7)	10 (7)	10 (7)
Extremely likely	22 (7)	12 (8)	10 (7)
Napper self-perceived HIV risk, mean (SD)^c^	19.10 (5.39)	19.10 (5.47)	19.10 (5.33)
HIV knowledge score, mean (SD)^d^	9.25 (3.01)	9.35 (2.79)	9.15 (3.22)
PrEP^e^ knowledge score, mean (SD)^f^	3.69 (4.02)	3.99 (4.01)	3.40 (4.02)
Ever used PrEP, n (%)			
No	244 (82)	123 (83)	121 (81)
Yes	18 (6)	9 (6)	9 (6)
Do not know	37 (12)	17 (11)	20 (13)
PrEP intention score, mean (SD)^g^	6.32 (2.02)	6.23 (2.01)	6.41 (2.03)

a GED: General Education Degree.

b N/A: not applicable.

c The Napper self-perceived risk score was calculated based on responses to 8 items with possible scores ranging from 8 to 43, where 8 represents the lowest possible self-perceived risk and 43 the highest self-perceived risk.

d The HIV knowledge score was calculated based on true or false responses to 16 items with possible scores ranging from 0=all incorrect responses to 16=all correct responses.

e PrEP: pre-exposure prophylaxis.

f The PrEP knowledge score was calculated based on true, false, or do not know responses to 13 items with possible scores ranging from 0=all incorrect responses to 13=all correct responses.

g The PrEP intention score was calculated based on responses to 3 items each with a possible score of 1‐4. Possible scores range from 3 to 12, with 3 representing the lowest level of PrEP intention and 12 representing the highest level.

At enrollment, 34% (101/299) reported being somewhat, very, or extremely likely to test for HIV in the next 6 months. The mean self-perceived risk score for HIV was 19.10 (SD 5.4) out of a possible 43, the mean HIV knowledge score was 9.25 (SD 3.01) out of a possible 16, and the mean PrEP knowledge score was 3.69 (SD 4.02) out of a possible 13. In total, 6% (18/299) of participants had ever used PrEP, and the mean PrEP intention score was 6.32 (SD 2.02) out of a possible 12. Participants in the 2 treatment arms were similar based on baseline characteristics.

### Participant Self-Reported Sexual and HIV Risk Behaviors

Participants reported a median of 3 (IQR 1‐7) lifetime sexual partners, and 60% (178/299) had been sexually active within the last month, with most engaging in oral sex, followed by vaginal and anal sex (Table 2). A majority (256/299, 86%) reported engaging in either unprotected oral, vaginal, or anal sex. About one quarter (72/299, 24%) of participants did not know their sexual partner’s HIV status, and 69% (206/299) reported that their recent sex partner was a primary partner. In total, 10% (30/299) of participants reported ever being diagnosed with an STI, with chlamydia being the most commonly diagnosed STI (19/30, 63%), followed by gonorrhea (5/30, 17%). The mean condom self-efficacy score was 6.45 (SD 2.69) out of a possible 20, which is the highest level of self-efficacy. Sexual and HIV risk behaviors were similar across arms.

**Table 2.** Participant self-reported sexual behaviors stratified by study arm.

	Overall (n=299)	Control app (n=149)	Game intervention (n=150)
Lifetime partners, median (IQR)	3.00 (1.00-7.00)	3.00 (1.00-6.00)	3.00 (1.00-7.00)
Last time sexually active, n (%)			
<1 month	178 (60)	87 (58)	91 (61)
1‐3 months	57 (19)	31 (21)	26 (17)
>3 months	64 (21)	31 (21)	33 (22)
Type of sexual activity engaged in, n (%)			
Oral (receptive)	264 (88)	127 (85)	137 (91)
Oral (insertive)	228 (76)	118 (79)	110 (73)
Vaginal (receptive)	147 (49)	68 (46)	79 (53)
Vaginal (insertive)	156 (52)	78 (52)	78 (52)
Anal (receptive)	65 (22)	40 (27)	25 (17)
Anal (insertive)	57 (19)	30 (20)	27 (18)
Other	4 (1)	2 (1)	2 (1)
Do not know	3 (1)	3 (2)	0 (0)
Unprotected sex	256 (86)	127 (85)	129 (86)
Sexual activity risk, n (%)			
Sex partner injects drugs	1 (0)	1 (1)	0 (0)
Sex partner infected with HIV	1 (0)	1 (1)	0 (0)
Sex partner HIV status unknown	72 (24)	35 (24)	37 (25)
None of the above	226 (76)	113 (76)	113 (75)
Engaged in exchange sex (yes), n (%)	7 (2)	2 (1)	5 (3)
Recent sex partner, n (%)			
Primary partner	206 (69)	103 (69)	103 (69)
Steady, nonprimary partner	18 (6)	10 (7)	8 (5)
Casual partner	65 (22)	35 (24)	30 (20)
Anonymous partner	7 (2)	0 (0)	7 (5)
Other	3 (1)	1 (1)	2 (1)
Do not know recent sex partner’s HIV status, n (%)	74 (25)	37 (25)	37 (25)
Ever diagnosed with an STI^a^, n (%)			
No	262 (88)	131 (88)	131 (87)
Yes	30 (10)	16 (11)	14 (9)
Do not know	5 (2)	1 (1)	4 (3)
Prefer not to answer	2 (1)	1 (1)	1 (1)
Type of STI diagnosis (n=30), n (%)			
Chlamydia	19 (63)	9 (56)	10 (71)
Gonorrhea	5 (17)	4 (25)	1 (7)
Trichomoniasis	1 (3)	1 (6)	0 (0)
Syphilis	1 (3)	1 (6)	0 (0)
Genital herpes	0 (0)	0 (0)	0 (0)
Genital warts	2 (7)	1 (6)	1 (7)
Pelvic inflammatory disease	0 (0)	0 (0)	0 (0)
Other	5 (17)	3 (19)	2 (14)
Condom self-efficacy score, mean (SD)^b^	6.45 (2.69)	6.26 (2.36)	6.64 (2.99)

a STI: sexually transmitted infection.

b Condom self-efficacy score was calculated based on responses to 4 items, with responses on a 5-point scale. The possible range of scores is 4=lowest self-efficacy to 20=highest self-efficacy.

### Primary Efficacy and Secondary Outcome Analyses

In our analysis assessing the efficacy of the game intervention compared to the control app for our primary outcome of HIV testing, 26% (32/150) of game participants versus 38% (48/149) of control participants reported being tested for HIV within 6 months. Game participants were 32% less likely than control arm participants to be HIV tested at 6 months (RR 0.68, 95% CI 0.47‐0.98; Table 3). With respect to intention to get tested in the next 6 months and intent to start PrEP, there were no statistically significant differences by study arm.

**Table 3.** Analysis of main and secondary outcomes^a^ at 6 months.

	Control app, n (%)	Game intervention, n (%)	Game versus app, RR^b^ (95% CI)	*P* value	Missing, n
HIV testing	48 (38)	32 (26)	0.68 (0.47-0.98)	.04^c^	51
Intent to test	81 (62)	74 (58)	0.94 (0.77-1.15)	.56	41
PrEP^d^ initiation	14 (11)	11 (9)	0.80 (0.38-1.69)	.56	53

a All outcomes are dichotomous.

b RR: relative risk.

c Statistical significance.

d PrEP: pre-exposure prophylaxis.

When we examined selected secondary outcomes over time, we found that with respect to HIV knowledge, the treatment effect varied over time (LRT *P*<.001) such that there was a statistically significant difference in mean knowledge scores at 3 (*P*=.003) and 6 months (*P*=.02), with the game arm scoring nearly one point (0.99, 95% CI 0.33‐1.65) higher than the control app arm at month 3 and 0.81 (95% CI 0.14‐1.48) points higher at month 6. There were no statistically significant differences for other outcomes including PrEP knowledge, intent to test, condom self-efficacy, self-perceived risk, or PrEP intention (Table 4).

**Table 4.** Analysis of additional secondary outcomes: knowledge and intent scores.

	Likelihood ratio test, *P* value	Model-based mean difference in scores (game vs app)							
		Baseline		1 month		3 months		6 months	
		Mean difference (95% CI)	Wald *P* value	Mean difference (95% CI)	Wald *P* value	Mean difference (95% CI)	Wald *P* value	Mean difference (95% CI)	Wald *P* value
HIV knowledge	*<.001* ^a^	−0.20 (−0.83 to 0.43)	.54	0.12 (−0.53 to 0.76)	.72	0.99 (0.33 to 1.65)	*.003*	0.81 (0.14 to 1.48)	*.02*
PrEP^b^ knowledge	*<.002*	−0.59 (−1.39 to 0.22)	.15	0.52 (−0.31 to 1.34)	.22	0.82 (−0.02 to 1.66)	.06	0.41 (−0.44 to 1.26)	.34
Intent to test	.32	0.00 (−0.30 to 0.30)	.99	0.00 (−0.30 to 0.30)	.99	0.00 (−0.30 to 0.30)	.99	0.00 (−0.30 to 0.30)	.99
Condom self-efficacy	.64	0.15 (−0.17 to 0.48)	.36	0.15 (−0.17 to 0.48)	.36	0.15 (−0.17 to 0.48)	.36	0.15 (−0.17 to 0.48)	.36
Self-perceived risk	.63	−0.34 (−1.45 to 0.77)	.55	−0.34 (−1.45 to 0.77)	.55	−0.34 (−1.45 to 0.77)	.55	−0.34 (−1.45 to 0.77)	.55
PrEP intention	.78	0.14 (−0.27 to 0.56)	.50	0.14 (−0.27 to 0.56)	.50	0.14 (−0.27 to 0.56)	.50	0.14 (−0.27 to 0.56)	.50

a Italics format indicates that *P* values <.05 are statistically significant.

b PrEP: pre-exposure prophylaxis.

### Ancillary Analyses

We performed the Little missing completely at random (MCAR) test, restricting the data to numerical variables, and the 3 primary outcome variables coded as 0‐1 binary variables. There were 15 missing data patterns, and the test statistic value was 270.0 with corresponding degrees of freedom of 220 and *P* value of .01, indicating adequate evidence against the MCAR assumption. We conducted sensitivity analyses of our primary and secondary outcomes accounting for missing data using multiple imputation, which is valid under the less restrictive missing at random assumption than MCAR, as well as stratified by sexual orientation (Tables S1-S4 in Multimedia Appendix 1). The estimated RR for the primary outcome of HIV testing at 6 months based on multiple imputation (0.68) was the same as that from the complete case analysis shown in Table 3; however, the corresponding *P* value is .05. Results for the intention to get tested in the next 6 months and intent to start PrEP were similar in the complete case and multiple imputation analyses.

Among participants identifying as heterosexual, the PrEP intention score increased on average 0.53 points (95% CI 0.05‐1.00) in the game intervention arm compared to the control app arm across all study time points (*P*=.03; Table S3 in Multimedia Appendix 1). Additionally, at the 3-month time point, game participants identifying as heterosexual had a mean HIV knowledge score that was 1.52 points higher (95% CI 0.49‐2.56) compared to the control arm (*P*<.01; Table S4 in Multimedia Appendix 1).

### Game and Control App Usability Data

When assessing game intervention and control app usability data at 1 and 3 months, we found that at 1 month, participants in the game arm compared to the control had lower mean SUS scores (67.9, SD 14.22 vs 72.8, SD 12.26; *P*<.01), yet had statistically significantly higher MAKE and overall scores (all *P*<.01; Table S5 in Multimedia Appendix 1). At the 3-month follow-up, game arm participants had lower SUS scores versus control, but retained higher scores on all components of the MAKE measures (all *P*<.01). There were no statistically significant differences in overall game-app satisfaction at 1 month or 3 months when comparing across arms.

### Game Intervention and Control App Paradata

The median number of times that a participant engaged with the game was 27 (IQR 7.4‐64.0) minutes over the 3-month intervention period (Table S6 in Multimedia Appendix 1). When assessing game intervention progression through the various stages of the game, 47% (70/150) of participants progressed up to 25% of the way through the game; 16% (24/150) progressed through the entire game content. The median number of HIV learning content items seen was 10 (IQR 5‐15) out of a possible 18 items. The median number of times that a participant opened a map to look for a provider was 2 (IQR 0‐3). For participants in the control app arm, the median number of app openings was 4 (IQR 2‐6) over the 3-month period; and the median number of URL clicks to access additional content was 0 (IQR 0‐1).

## Discussion

### Interpretation

Using primarily online social media recruitment, we were able to recruit and retain a diverse population of adolescents and young adults at risk for HIV infection living in the Washington, DC area for a randomized controlled trial of an interactive educational gaming intervention compared to an educational control app. While we hypothesized that the game intervention would be more effective in increasing HIV testing among adolescents and young adults after a 3-month exposure period, we found the gaming app was 32% less effective than the control condition in increasing HIV testing at the end of 6 months. Additionally, secondary data analyses also indicated that HIV testing intentions and intentions to start PrEP were not higher in the gaming group compared to the control group at 6 months. We did, however, observe a minimal yet statistically significantly higher HIV knowledge score among game intervention participants at 3 and 6 months, suggesting both short-term and sustained effects and that the interactive game intervention may have been better at delivering information on HIV to participants compared to the control. This effect persisted when stratifying by sexual orientation, with heterosexual participants having statistically significantly higher HIV knowledge scores at the 3-month time point and higher PrEP intention scores at all time points compared to the control arm. Finally, there were no statistically significant differences for secondary outcomes such as PrEP knowledge, intent to test for HIV, condom self-efficacy, and self-perceived risk.

Our findings are consistent with other HIV testing and prevention serious gaming app-based interventions focused on adolescents and young adults with respect to increases in HIV knowledge. For example, the MyPEEPS mobile randomized trial, which focused on reducing sexual risk among young men who have sex with men, found no differences in HIV testing or PrEP uptake, which is consistent with our results [67]. On the other hand, PlayTest!, a school-based randomized trial, found improvements in HIV testing attitudes and intentions, knowledge, and self-efficacy [68]. Several studies have reported preliminary improvements in sexual behavior knowledge and risk perception from pilot testing [37,69-71]; however, efficacy trial results are not available for these studies. As for our secondary outcomes, several other studies, including the Tumaini trial in Kenya, which was a community-level 2-arm randomized trial aimed at delaying the age of sexual debut and increasing condom use among persons aged 13‐14 years [72], found significant effects on condom self-efficacy, as have other mobile gaming apps [69].

Possible explanations for the lack of efficacy of the intervention game to increase HIV testing may be related to the usability and acceptability of the gaming intervention and study design used. Although usability of the game was considered above average and higher MAKE scores were observed among the game intervention participants, we did not observe any statistically significant differences in satisfaction across the 2 study arms. The difference in usability scores across the 2 arms may be explained by the fact that the game was complex, had multiple steps, and was intentionally more interactive than the control app [73,74]. For example, intervention participants had to learn to play the game, whereas control arm participants only had to navigate a static informational app. Further, since control app satisfaction was also relatively high, the control app may not have represented a real-world placebo condition. However, it may also suggest that a lower level “intervention” such as our established control materials may be as effective as an interactive game. Other possibilities may be explained by the sample composition, where baseline participants already had a moderate level of HIV knowledge, and almost one-third reported already being likely to test for HIV in the next 6 months. The finding of higher PrEP intention among heterosexual-identifying participants is reassuring that the game content may have resulted in increased PrEP education and awareness among these participants who may not always be the focus of HIV prevention and PrEP messaging [8-11].

To increase the ability to measure the efficacy of the game, future iterations of the intervention might include a 3-arm trial with an observational group compared to the control app and the game intervention [75,76]. Alternatively, it was unclear if the duration of the game intervention may have been too short to demonstrate an effect; therefore, allowing participants continued access to the intervention components after the designated intervention period could determine if the intervention is delivered more effectively as a continuously available resource in a real-world setting versus being delivered over a specified and limited period of time. Finally, adding additional components to the game such as the ability to order home-based HIV and STI test kits [77,78] may have resulted in increased game use and possible efficacy results.

The game paradata also provided additional data to assist with interpretation of our findings. For example, it may be that the interactive game was not sufficiently engaging for our participants. Paradata found that game participants had relatively limited use at just under 30 minutes, and only 16% progressed through all of the game content with exposures to a median of 10 of 18 HIV educational items. Control app use was also limited, with a median of 4 openings and few to no clicks for supplemental information. Additional analyses by level of game use or content viewing may provide further information on the characteristics of participants who received various levels of exposure to the HIV content from the game and the potential dose-response effect on the primary and secondary outcomes [59,60].

Qualitative interviews were also conducted among a purposeful sample of game participants. These interviews are currently being analyzed and will provide valuable information to help elucidate game intervention components and features that were most effective and how the game can be improved upon to more effectively deliver HIV prevention and testing information to adolescents and young adults.

Despite the lack of efficacy of the intervention, we were successful in recruiting and retaining a diverse cohort of adolescents and young adults with respect to race, ethnicity, and sexual orientation. Importantly, 6-month retention ranged from 80% to 88%, and more than half of our sample self-identified as lesbian, gay, bisexual, or other sexual orientation, and 38% identified as non-Hispanic Black or Hispanic. Our retention and participant diversity mirror that of similar interventions, which have also recruited racially and ethnically diverse groups of adolescents and young adults and had high retention rates [67]. Given that racial, ethnic, and sexual minority groups account for the highest proportions of new HIV diagnoses younger than 25 years of age [1], it was essential that we were able to include and consistently engage these individuals in testing our HIV prevention intervention. While we sought to recruit participants as young as 13 years of age in our study and tailored our social media advertisements to recruit younger participants, this proved more challenging, with only 8 participants younger than 18 years of age. At the time of study recruitment, several social media companies (eg, Meta) had restrictions placed that limited advertisements to persons younger than 18 years of age [79], and while we had over 1 million advertisement impressions from potential participants younger than 18 years of age, only 0.61% clicked on the advertisements. Moreover, of those 13-17 years of age who completed a screener (n=70), a total of 48 (69%) were ineligible for the study, as they had never been sexually active. Improved efforts to reach this younger age group are necessary and may include the delivery of school-based interventions, overall support for life skills development, educational videos, and clinic-based interventions that are accessible, relatable, and individually tailored to this younger age group [21,23,29,77,78,80,81]. While 6-month retention differed across the 2 study arms (80% game intervention vs 88% control arm), we do not think that this affected our results, as there were no baseline differences in randomization between the 2 groups, and study retention procedures were the same across the 2 study arms. This difference in retention may be partially explained by game fatigue, where participants tire of the existing game content and stop playing as much. Future iterations of the game could include periodically releasing new game content to increase participant engagement or inclusion of reward systems (eg, badges and prizes) to boost retention and increase access to HIV prevention educational material [82,83].

### Limitations

Limitations of our study include that we were only able to include English-speaking participants who lived in the DC metropolitan region and that we did not recruit a large enough sample younger than 18 years of age to conduct further stratification by age, thereby limiting the representativeness and generalizability of our findings. Study recruitment at clinical sites was limited due to the COVID-19 pandemic and subsequent operational changes at the clinic level. Had recruitment included clinic-attending adolescents and young adults, it may have resulted in recruitment of more participants younger than 18 years of age. The game was created to appeal to both adolescents and young adults; however, given the limited number of participants younger than 18 years of age, we do not know if it would have been more effective with this younger age group since design choices made for adolescents may not necessarily have the same appeal to young adults. Future intervention testing should consider different versions of the game based on age and more purposeful sampling of younger age groups, as they tend to face more barriers to accessing HIV education and testing than those older than 18 years of age [17,46,84]. Additionally, testing the intervention in regions of the United States that have different policies, laws, and levels of access to HIV preventive care than the DC area would also increase the generalizability and potential reach of our intervention, particularly in areas where HIV education may not be occurring in schools or access to HIV testing is restricted in adolescents and young adults [85]. Despite these limitations, strengths include successful social media recruitment with limited numbers of fraudulent participants and high retention rates among an often-transient population.

In conclusion, to our knowledge, very few other life-simulation apps focused on HIV testing among both adolescents and young adults, regardless of sexual orientation, with integration of HIV testing and prevention features have been rigorously tested for efficacy in the United States to date. Prior studies that have looked at increasing HIV testing uptake and knowledge are feasibility and pilot studies [32], have focused solely on young men who have sex with men [86], have been conducted outside of the United States [72], or have focused on PrEP adherence rather than PrEP education [87]. Moreover, results from large, randomized trials are limited [37,39,68,70,82,87], allowing our study to add to this important and emerging body of research.

In this study, we sought to determine if an interactive life-simulation approach to delivering HIV testing and prevention information would be more effective than an informational control app. We found that the interactive game was not more efficacious, and our “active” control app may be as good at engaging young adults in HIV prevention activities. Given the limited number of participants ages 13 to 17 years, additional testing of the intervention is needed to determine its potential efficacy among this younger age group. Nevertheless, the game intervention was able to increase HIV knowledge and PrEP intention among heterosexual adolescents and young adults, indicating that it was able to effectively deliver information to increase their exposure to HIV prevention and interventions. Our novel and early findings of observed improvement in PrEP intention add to the limited body of literature on the role of interactive gaming interventions in HIV prevention and PrEP specifically, and among all adolescents and young adults and not just young men who have sex with men [31,87,88]. Digital interactive game-based technologies are regularly used by adolescents and young adults, and their ability to deliver feasible, acceptable, scalable, and affordable HIV interventions is yet to be fully realized [31,88,89]. Thus, additional research and innovative approaches are needed to scale technology-based interventions to increase engagement in HIV testing and knowledge and PrEP intention and knowledge for all adolescents and young adults at risk for HIV acquisition so that they can be applied and shown to be effective in real-world settings.

## Supplementary material

10.2196/82297 Multimedia Appendix 1 Multiple imputation sensitivity analysis of main and secondary outcomes and stratified by sexual orientation.

10.2196/82297 Checklist 1 CONSORT checklist.

## Abbreviations

CDC: Centers for Disease Control and Prevention
CONSORT: Consolidated Standards of Reporting Trials
CONSORT-eHEALTH: Consolidated Standards of Reporting Trials of Electronic and Mobile Health Applications and Online Telehealth
IRB: institutional review board
LRT: likelihood ratio test
MAKE: Motivation, Attitude, Knowledge, and Engagement
MCAR: missing completely at random
PrEP: pre-exposure prophylaxis
RR: relative risk
STI: sexually transmitted infection
SUS: System Usability Score
