# Supplementary material for: Efficacy of an Interactive Life Simulation Gaming App on Improving HIV Testing Uptake Among Adolescents and Young Adults at Risk for HIV: Results of a Randomized Controlled Trial
Source: JMIR Serious Games. 2026 Sep 4;14:e82297. doi: 10.2196/82297 (PMC13548308; doi:10.2196/82297)
Supplement: Supplementary file 2 [file games-v14-e82297-s001.docx]

**Multiple Imputation Sensitivity Analysis of Main and Secondary Outcomes: Methods**

As a sensitivity analysis, we analyzed the primary and secondary outcomes after multiple imputation. Missing values for the primary and secondary outcomes at 6 months were imputed based on random forest models implemented by the ranger function via the MICE package in R. Random forest classifiers (for the binary outcomes) were built using all clinical, demographic, and sexual risk measures shown in Tables 1 and 2 as well as treatment arm, all scores collected at 1, 3, and 6 months, and the outcomes collected at 1, 3, and 6 months (omitting the 6-month outcome being imputed). Missing values for any of the variables just listed were also imputed using either regression random forest (for numerical variables) or classification random forest (for categorical variables) as well. Aside from building 500 trees for each random forest model, we utilized the defaults for all other hyperparameters of the ranger function. We created 30 imputed data sets based on 10 iterations of the MICE procedure for each data set and combined the results across the imputed data sets using Rubin’s rules to obtain valid estimates for risk ratios and their corresponding 95% confidence intervals and p-values.

**Table S1**. Sensitivity Analysis of Main and Secondary Outcomes* (Multiple Imputation)

|  | **RR (95% CI) p-value** |
| --- | --- |
|  | Game v. App |
| HIV Testing | 0.68 (0.46, 1.01) p = 0.05 |
| Intent to Test | 0.96 (0.78, 1.19) p = 0.73 |
| PrEP Initiation | 0.78 (0.37, 1.66) p = 0.52 |

1. All outcomes are dichotomous.

**Table S2**. Analysis of Main and Secondary Outcomes^1^ Stratified by Sexual Orientation

|  | **Heterosexual** | | | | **Non-Heterosexual** | | | |
| --- | --- | --- | --- | --- | --- | --- | --- | --- |
|  | **Control App** | **Game Intervention** | **RR (95% CI) p-value** | **Missing** | **Control App** | **Game Intervention** | **RR (95% CI) p-value** | **Missing** |
|  | n (%) | n (%) | Game v. App | n | n (%) | n (%) | Game v. App | n |
| HIV Testing | 21 (37.5) | 12 (21.4) | 0.57 (0.31, 1.05) p = 0.06 | 29 | 27 (39.1) | 20 (29.9) | 0.76 (0.48, 1.22) p = 0.26 | 22 |
| Intent to Test | 33 (55.9) | 32 (55.2) | 0.99 (0.71, 1.36) p = 0.93 | 24 | 48 (66.7) | 42 (60.9) | 0.91 (0.71, 1.17) p = 0.47 | 17 |
| PrEP Initiation | 3 (5.5) | 2 (3.6) | 0.67 (0.12, 3.84) p = 0.65 | 31 | 11 (15.9) | 9 (13.4) | 0.84 (0.37, 1.9) p = 0.68 | 22 |

1. All outcomes are dichotomous.

**Table S3**. Analysis of Additional Secondary Outcomes: Knowledge and Intent Scores Stratified by Sexual Orientation^1^

| **3a** | **Heterosexual** | | **Non-Heterosexual** | |
| --- | --- | --- | --- | --- |
| **Outcome** | **Likelihood Ratio Test p-value^2^** | **Model-Based Mean Difference in Scores (Game – Control App) (95% CI) and Wald p-value** | **Likelihood Ratio Test p-value^2^** | **Model-Based Mean Difference in Scores (Game – Control App) (95% CI) and Wald p-value** |
| HIV Knowledge | **0.002** | **See Table S4.** | 0.12 | 0.29 (-0.35, 0.93)  p = 0.38 |
| PrEP Knowledge | 0.17 | 0.11 (-0.77, 0.99)  p = 0.80 | **0.006** | **See Table S4.** |
| Intent to Test | 0.05 | 0.23 (-0.15, 0.61)  p = 0.24 | 0.99 | -0.20 (-0.64, 0.25)  p = 0.39 |
| Condom Self-Efficacy | 0.17 | 0.09 (-0.44, 0.62)  p = 0.74 | 0.85 | 0.21 (-0.19, 0.62)  p = 0.30 |
| Self-Perceived Risk | 0.92 | 0.47 (-1.00, 1.94)  p = 0.53 | 0.73 | -1.03 (-2.53, 0.47)  p = 0.18 |
| PrEP Intention | 0.68 | 0.53 (0.05, 1.00)  **p = 0.03** | 0.77 | -0.20 (-0.85, 0.45)  p = 0.56 |

1. Estimated effects shown in this table are assumed constant over time since the Likelihood Ratio Tests for interaction between time and treatment arm are greater than 0.05.

2. P-values <0.05 are bolded to indicate statistical significance.

**Table S4.** Analysis of Additional Secondary Outcomes: Knowledge and Intent Scores Stratified by Sexual Orientation^1^

| **3b** |  | **Model-Based Mean Difference in Scores (Game – Control App) (95% CI) and Wald p-value** | | | |
| --- | --- | --- | --- | --- | --- |
| **Outcome** | **Likelihood Ratio Test p-value^2^** | **Baseline** | **1 Month** | **3 Months** | **6 Months** |
| HIV Knowledge Heterosexual | **0.002** | -0.26 (-1.23, 0.72)  p = 0.61 | 0.21 (-0.80, 1.21)  p = 0.69 | 1.52 (0.49, 2.56)  **p = 0.004** | 0.81 (-0.23, 1.84)  p = 0.12 |
| PrEP Knowledge  Non-Heterosexual | **0.006** | -0.62 (-1.71, 0.47)  p = 0.26 | 0.58 (-0.52, 1.69)  p = 0.30 | 0.84 (-0.29, 1.97)  p = 0.14 | 0.78 (-0.36, 1.92)  p = 0.18 |

1. Estimated effects shown in this table are assumed constant over time since the Likelihood Ratio Tests for interaction between time and treatment arm are greater than 0.05.

2. P-values <0.05 are bolded to indicate statistical significance.

**Table S5. Game Intervention and Control App Usability By Study Follow-up Time Period**

|  | **1-Month Survey** | | | **3-Month Survey** | | |
| --- | --- | --- | --- | --- | --- | --- |
|  | **Control App** | **Game Intervention** | **p-value** | **Control App** | **Game Intervention** | **p-value** |
|  | n = 141 | n = 137 |  | n = 132 | n = 124 |  |
|  | Mean (SD) | Mean (SD) |  | Mean (SD) | Mean (SD) |  |
| System Usability Score^1^ | 72.75 (12.26) | 67.94 (14.22) | **0.003** | 70.02 (13.68) | 64.92 (14.39) | **0.004** |
| Game-App Satisfaction^2^ | 21.58 (4.38) | 21.52 (4.74) | 0.91 | 21.06 (5.17) | 21.52 (4.69) | 0.45 |
| MAKE Motivation^3^ | 39.74 (9.36) | 46.10 (8.59) | **<0.001** | 39.34 (10.87) | 47.10 (8.57) | **<0.001** |
| MAKE Attitude^3^ | 23.41 (5.87) | 25.91 (4.92) | **<0.001** | 22.86 (6.80) | 26.26 (5.19) | **<0.001** |
| MAKE Knowledge^3^ | 25.44 (5.36) | 26.41 (4.96) | 0.12 | 24.63 (6.81) | 26.73 (5.02) | **0.006** |
| MAKE Engagement^3^ | 15.35 (4.59) | 18.62 (3.42) | **<0.001** | 15.17 (4.83) | 18.65 (3.77) | **<0.001** |
| MAKE Total^3^ | 103.94 (23.20) | 117.04 (19.98) | **<0.001** | 101.99 (27.95) | 118.75 (20.88) | **<0.001** |

1. The System Usability Score (SUS) is based on responses to 10 items with 5 response options. Possible scores range from 0-100 applying the scoring criteria with a mean score of 68 or higher indicating above average usability.
2. Game and App satisfaction scores were based on responses to 8 items using Likert scales ranging from 1 to 4 with a total possible range of scores from 8-32.
3. We used relevant measures from the MAKE Scale. These included 4 components of game-based learning: motivation (13 items; score up to 65), attitude (7 items; max score of 35), knowledge (7 items; max score of 35) and engagement (5 items; max score of 25)). Each item is rated on a 5-point Likert scale ranging from 1= “strongly disagree” to 5 “strongly agree”.
4. P-values <0.05 are bolded to indicate statistical significance.

**Table S6**. **Summary of Game Intervention and Control App Paradata, N=309**

| **Game Paradata** | **n = 150** |
| --- | --- |
| **Cumulative Time Engaging with Game** (minutes) (median [IQR]) | 27.43 [7.40, 64.01] |
| **Game Progress** n(%) |  |
| Build Avatar: 0% Progress | 6 (4.) |
| Gym: 1-25% Progress | 70 (46.7) |
| Waiting Room: 26-50% Progress | 18 (12) |
| Community: 51-75% Progress | 24 (16) |
| Nightclub: 76-99% Progress | 8 (5.3) |
| DJ: 100% Progress | 24 (16) |
| **HIV Learning Content Items Seen** (median (IQR)) | 10.00 (5.00, 15.00) |
| **Times Provider Map Opened** (median (IQR)) | 2.00 (0.00, 3.00) |
|  | |
| **Control App Paradata** | **n = 149** |
| **App Openings** (median (IQR)) | 4.00 [2.00, 6.00] |
| **URL Openings** (median (IQR)) | 0.00 [0.00, 1.00] |
